# High-throughput computational X-ray absorption spectroscopy

**DOI:** 10.1038/sdata.2018.151

**Published:** 2018-07-31

**Authors:** Kiran Mathew, Chen Zheng, Donald Winston, Chi Chen, Alan Dozier, John J. Rehr, Shyue Ping Ong, Kristin A. Persson

**Affiliations:** 1 Department of Materials Science, University of California Berkeley, Berkeley, CA 94720, USA; 2 Department of Nanoengineering, University of California San Diego, La Jolla, CA 92093, USA; 3 Energy Technologies Area, Lawrence Berkeley National Laboratory, Berkeley, CA 94720, USA; 4 Division of Applied Research and Technology, National Institute for Occupational Safety and Health, Centers for Disease Control, Cincinnati, OH 45226, USA; 5 Department of Physics, University of Washington, Seattle, WA 98195, USA

**Keywords:** Atomistic models, Materials science, Scientific data

## Abstract

X-ray absorption spectroscopy (XAS) is a widely-used materials characterization technique. In this work we present a database of computed XAS spectra, using the Green's formulation of the multiple scattering theory implemented in the FEFF code. With more than 500,000 K-edge X-ray absorption near edge (XANES) spectra for more than 40,000 unique materials, this database constitutes the largest existing collection of computed XAS spectra to date. The data is openly distributed via the Materials Project, enabling researchers across the world to access it for free and use it for comparisons with experiments and further analysis.

## Background & Summary

A crucial step in the process of novel materials discovery is the characterization of the synthesized material. There exists a wide array of tools and spectroscopic techniques that are used in the material identification process, e.g. X-ray diffraction (XRD), X-ray emission spectroscopy (XES), and X-ray absorption spectroscopy (XAS). XAS is widely-employed in the characterization of the local structural environment surrounding select elements within a material.

Great progress has been made over the past few years in the development of laboratory-based X-ray spectrometers for high-resolution x-ray absorption near edge structure (XANES) and X-ray emission spectroscopy (XES)^1^. The availability of relatively inexpensive laboratory-based XAFS system (http://easyxafs.com/) and third generation synchrotron facilities^2^ have established the groundwork for the broad application of high-resolution XAS in characterization of materials. On the other hand, modern computational resources and methodologies have reached a level of maturity and efficiency to complement as well as to fast-track new discoveries. In the case of XAS, a variety of theoretical frameworks including time-dependent density-functional theory (TDDFT)^3,4^, multiple-scattering^5^, and Bethe-Salpeter equation (BSE) based approaches^6^ have been implemented, each exhibits its advantages and drawbacks. Leveraging spectroscopic simulation software with large crystal structure databases enables the computation of a large number of reliable theoretical spectra corresponding to well-defined crystal structures^7^, providing a broad reference dataset with clean unique structural fingerprints that can be used for identification purposes. With the help of carefully crafted software tools, these computations can be performed in a high-throughput fashion and can be used to scan the structural phase space for novel materials. In addition, through proper integration with modern database tools, these scans can be saved for future use and leveraged for training machine learning algorithms to assist the characterization process. Some examples of such publicly available spectroscopic database are the EELS Data Base^8^, a compilation of valence and core-loss spectra from EELS and XAS experiments containing 271 spectra that covers 39 elements of the periodic table, and XCOM (https://www.nist.gov/pml/xcom-photon-cross-sections-database), which provides photon cross sections for scattering, photoelectric absorption and pair production, as well as total attenuation coefficients, for any element, compound or mixture. Other existing XAS databases^9,10^, i.e. https://www.cat.hokudai.ac.jp/catdb/ and http://cars.uchicago.edu/xaslib, covering a few hundred spectra, are hosted across the world and serve as valuable references for analysis.

The FEFF framework affords relatively inexpensive calculations compared to other approaches and requires minimum adjustable parameters. It provides an efficient means of generating high quality XAS spectra for a larger amount of chemical systems and structures. Hence, in our study, we selected the FEFF9 (ref. 5) program for the *ab initio* calculation of K-edge X-ray absorption near edge spectra (XANES). Using the parameter settings obtained from recent benchmarking work against experimental spectra^11^ and the FEFF workflow infrastructure available in the open source materials science workflow package Atomate^12^, we generate spectra of all the materials available in the publicly accessible and widely used materials database, Materials Project (MP)^7^.

A comprehensive database of computed XAS spectra enables comparison between different spectroscopic signatures across chemical systems and structures such that rapid determination of oxidation states, coordination environment, and other local atomic structure information can be obtained. Furthermore, using matching algorithms^11^ or other machine learning methods^13^, the data can be leveraged for on-the-fly characterization. Though the peak positions and amplitudes of the computational K-edge XANES spectral may exhibit differences compared to experimental spectra, theoretically computed XANES spectra provide sufficient information to identify oxidation state and coordination chemistry of the probe atom, and can be highly useful when experimental data are not available or scarce. For example, a previous study by Timoshenko *et al*.^14^ showed that *ab initio* XANES spectra provide excellent input data for training supervised machine learning models aimed at reconstructing metal catalyst structures from their experimental XANES. The current authors have also shown in a previous study^11^ that an ensemble-learned algorithm to match experimental K-edge XANES spectra in the EELS Data Base to computed spectra can achieve nearly 80% accuracy in identifying the correct oxidation state and coordination environment. In addition, the data is associated with download options and programmatic analyses tools for each structure in the Materials Project database, thereby making it accessible to the broader materials science community. Furthermore, the MP web application enables users to select spectra from the database, upload experimental spectra data and predict the material composition using the matching tool. To date, this is the largest computed XAS dataset available and it is still expanding.

The paper is organized as follows; first we briefly describe the XAS computation methodology as implemented in the FEFF code, and thereafter the high-throughput framework used in the generation of the spectra. We then describe the data storage and dissemination details, followed by the technical validation of the computational methodology and the high-throughput framework.

## Methods

### Theory

The K-edge XANES spectra were computed using the FEFF^5^ code which employs the Green's formulation of the multiple scattering theory to compute the spectra^5^. The X-ray absorption *μ* is computed in a manner similar to Fermi's golden rule when written in terms of the projected photoelectron density of final states or the imaginary part of the one-particle Green's function, G(*r,r′;E*). In terms of the Green's function, G(*r,r′;E*), the absorption coefficient, *μ*, from a given core level *c* is given by ref. 15.

(1)µ=−1πIm<c|ε⋅rG(r,r';E)ε⋅r|c>

with the Green's function, G(*r,r′;E*) given by

(2)G(r,r';E)=∑fΨ f(r)Ψf(r')*E−Ef+iΓ

where $\psi_{f}$ are the final states, with associated energies *E*_*f*_ and net lifetime Γ, of a one-particle Hamiltonian that includes an optical potential with appropriate core hole screening.

The FEFF code computes the full propagator *G* incrementally using matrix factorization and uses the spherical muffin-tin approximation for the scattering potential^15^. For a more detailed description, we direct the readers to the review paper by Rehr *et al*.^15^

### High-throughput Workflow

**Figure 1:**
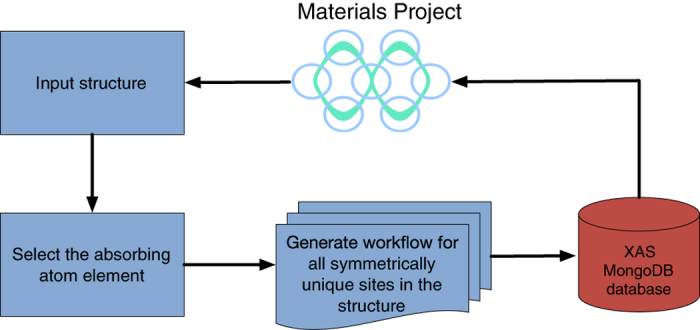
Schematic diagram of the high throughput framework employed in the generation of XAS spectra for the Materials Project.

For the high-throughput XAS spectra generation, we use the FEFF workflow (Fig. 1) available in the open source computational materials science workflow package Atomate^12^. Atomate provides a high-level interface to compose workflows using open source materials science softwares such as pymatgen^16^, FireWorks^17^ and Custodian (https://github.com/materialsproject/custodian).

Each FEFF calculation involves the following 3 steps:

- Selection of the absorbing site and the cluster of atoms to be included in the scattering calculations.
- Generation of the FEFF input files for each site and its surrounding atomic cluster.
- Execution of the FEFF binary on the generated input files.

As shown in Fig. 1, the workflow is initiated by importing a structurally optimized compound from MP. Each site in the downloaded structure is a possible absorbing center and FEFF calculation sequence must be initiated for each site. However, the number of calculations can be reduced by considering only the symmetrically unique sites in the structure. The FEFF input files for each such symmetrically unique absorbing site are generated subsequently and the FEFF binary is invoked on each input set. In the final step, the computed spectra from each calculation is inserted into a MongoDB database and disseminated via the Material Project (https://materialsproject.org/) website.

### Code availability

Except the for FEFF code, which is proprietary, all the other aforementioned packages used in the high-throughput XAS workflow are open source and can be found at https://github.com/materialsproject and https://github.com/hackingmaterials/atomate.

## Data Records

**Figure 2:**
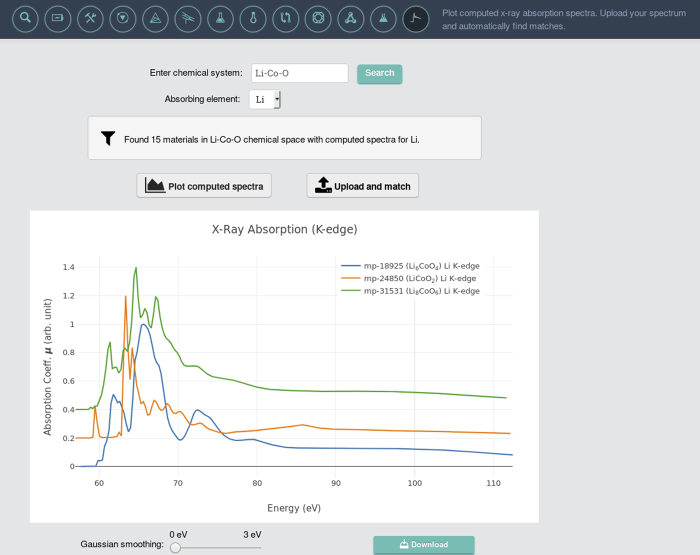
Screen shot of *XAS Matcher* web application. The web application is hosted at https://materialsproject.org/#apps/xas.

All the data described in this section can be found in the file, *xas.json.tgz* (Data Citation 1). The same data is also stored in the Materials Project database and is made freely available to the public. We also provide a user friendly web application called *XAS Matcher* (screenshot shown in Fig. 2) that enables user interaction with the computed data. The app can be reached at https://materialsproject.org/#apps/xas. Users can employ the app to search for computed XAS spectra, upload experimental spectra and find structures in the MP database whose computed spectra match that of the uploaded one. Details on spectra matching algorithm employed on Materials Project were published separately^11^.

### Data Representation

To date, spectra for more than half of the compounds(≈40000) in the Materials Project database are available, for all the symmetrically unique sites in each structure. Each structure dataset is stored in the database in the binary JavaScript Object Notation (BSON) format. The keys and respective descriptions are summarized in Table 1. Although the workflow yields separate spectra for each unique atomic site, the averaged absorption coefficient over all the sites in the structure with that element is presented on the MP website. This will facilitate comparison with experimental spectra, where the averaging over each element is unavoidable. However, the full data, e.g spectra for all unique sites, are available to the user for download and further analysis.

### Data Download

The spectral data as well as the input parameters used for the calculations can be downloaded either directly from the Material Project website or using the REST Application Programming Interface (API) available in pymatgen^18^. Data can be downloaded for each element in the selected structure. The downloaded spectrum is provided in a tab separated file format and includes the spectral data for all the symmetrically unique sites of the selected element in the structure. The standard XAS data interchange (XDI) format^19^ is also available for download, which can be directly imported into most existing XAFS data analysis programs^20^ for further detailed analysis.

## Technical Validation

### Verification of the default parameter settings for the workflow.

The workflow described above relies on the default FEFF input parameter settings to generate the K-edge XANES spectra in a high throughput fashion. In this section, we will briefly describe the major FEFF input parameters relevant to the calculation of the XANES spectra, the bench-marking procedure and sample validation cases against experimentally available XANES spectra.

FEFF9 is capable of achieving quantitative agreement with XAS experimental results with a minimal set of adjustable parameters. The development and implementation of parameter-free models within the FEFF9 code permit consistent calculations across different chemical systems and constitute the main advantage for high-throughput calculations. In the benchmarking process, we included 13 unique compounds and their corresponding high-quality K-edge XAS spectra available in the open EELS/XAS database^8^, supplemented by 6 experimental XANES spectra of V_2_O_5_, V_2_O_3_, VO_2_, LiNiO_2_, LiCoO_2_, and NiO from previous studies^21,22^. Compounds included in the earliest commentary^5^ of FEFF9 software were also evaluated. The benchmark compound dataset has a high structural diversity and covers a wide chemical space. Detailed benchmark information is provided in a previous publication^11^. For benchmark compounds that contain detailed structural information, we used structures from the Materials Project (https://materialsproject.org/) database that exhibit an optimized geometry with the same space group as the benchmark compound. For benchmark compounds without provided structural information, MP ground state structures with identical chemical compositions were used^11^.

The following input fields in FEFF9 were subjected to convergence and optimization tests:

- *Self-consistent field (SCF)*: The SCF card controls FEFF automated self-consistent potential calculations.The self-consistent potential calculation is required in the XANES calculation for the Fermi level *E*_0_ estimation. In the convergence test, we varied the number of atoms included in the self-consistent potential calculations through changing the **rfms1** value from 2 Å to 8 Å at 1 Å interval.
- *Full multiple scattering (FMS)*: The FMS card is required in the XANES calculation as the multiple scattering (MS) expansion's convergence might not be stable in the XANES calculation^5^. To identify the effect of **rfms** field on XANES calculation results, we varied the **rfms** value from 3 Å to 11 Å at 1 Å interval.
- *EXCHANGE*: The EXCHANGE card specifies the exchange correlation potential model used for XANES calculation. The Hedin-Lundqvist self-energy is chosen as previously recommended for most applications^23^.
- *COREHOLE*: The COREHOLE card is used for specifying how the core is treated during XANES calculation. The default choice in FEFF treats the core-hole interaction based on the Final State Rule (FSR), which could overestimate the strength of the core-hole and excludes the core-hole mixing effect^24^. To overcome these deficiencies and avoid possible break down of FSR for the L-shell metals^25^, the random phase approximation (RPA) is used to approximate the core-hole interactions in our high-throughput K-edge XANES calculations.

Through the benchmarking study, a set of optimized FEFF parameters were determined to achieve the best balance between the computational cost and convergence performance. The Pearson correlation coefficient is used to compare spectra calculated using different parameters. The Pearson correlation coefficient between two same energy grid spectra, *X*_*i*_ and *Y*_*i*_, is calculated using the following expression:

(3)SPearson(X,Y)=∑i=1D(Xi−X¯)(Yi−X¯)(∑i=1D(Xi−X¯)2)(∑i=1D(Yi−Y¯)2),

where *X*_*i*_ and *Y*_*i*_ are the corresponding absorption coefficients.

**Figure 3:**
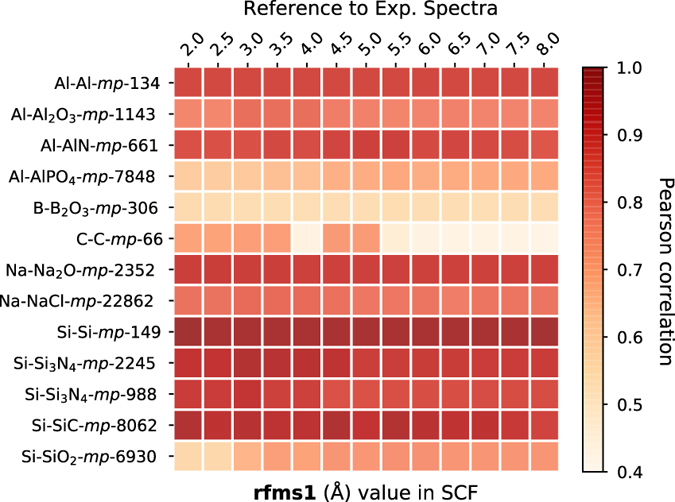
Benchmarking results of rfms1 parameter in the SCF card for K-edge XANES of various materials. Pearson correlation coefficients were calculated between spectra calculated at different rfms1 and the experimental reference provided by electron energy-loss spectroscopy (EELS) Data Base^8^.

**Figure 4:**
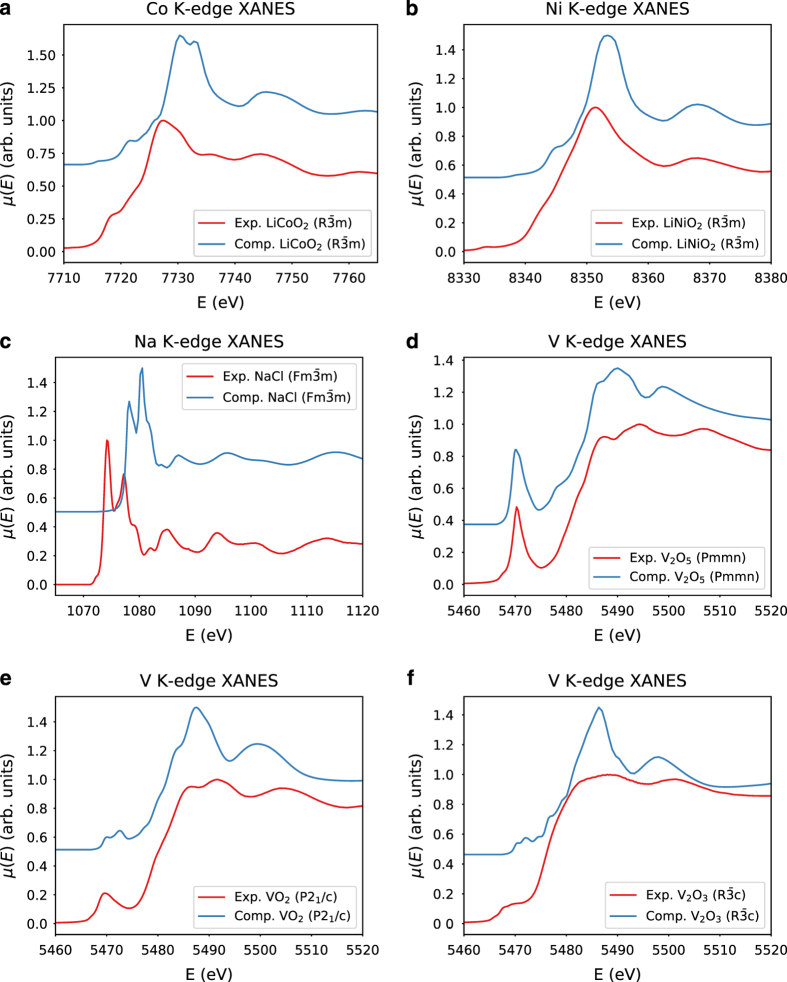
Sample comparisons of FEFF computed K edge XANES spectra with the corresponding experimental ones for six different compounds. Computational spectra are shifted upwards by 0.5. (**a**) *LiCoO*_2_, (**b**) *LiNiO*_2_, (**c**) *NaCl*, (**d**) *V*_2_*O*_5_, (e) *VO*_2_, (f) *V*_2_*O*_3_.

We noticed that the Pearson correlation coefficients between FEFF computed spectra and experimental spectra obtained from EELS Data Base are above 0.85 in general (see Fig. 3). For *C* and *B*_2_*O*_3_, the FEFF-computed spectra are not in good agreement with experimental spectra. Possible solutions include the adoption of other higher-level real-space full-potential multiple scattering theory or first principles approaches^26^, which are not ideal for high-throughput implementation due to their high computational cost. Figure 4 depicts some sample comparisons between the computed and the experimental K-edge XANES spectra. We note that the computed spectra match with that of the experimental ones only up to a constant shift in the energy. The computed K-edge XANES spectra of vanadium oxides given in Fig. 4d–f show a strong change in their first peak intensity. Reasonably good agreement between computational and experimental spectra was found.

## Usage Notes

We present a database of K-edge XANES spectra computed using FEFF. The data is made freely available to all researchers via the Materials Project(www.materialsproject.org). Users can also download the data using the REST API that is part of pymatgen. All the codes used to create the high throughput are made freely available at Github(https://github.com/materialsproject and https://github.com/hackingmaterials/atomate). We hope that the users will find the data to be useful and will find novel ways to employ the data to accelerate their research. One such use case would be using machine learning techniques to predict structures from the experimentally measured spectra.

For users of FEFF and the spectra resulting in this study, it should be noted that K-edge XANES spectra computed by FEFF are more accurate for the investigation of elements in the periodic table up to the fifth-row. For excitations in heavier elements, e.g., the rare earth elements and 5*d* elements, L-edge XANES spectra are primarily used. FEFF is also applicable for the simulation of L-edge XANES spectra, though in certain cases^27–29^ ground-state DFT methodologies need to be used for better agreement between computed spectra and experimental results. A detailed study of high-throughput FEFF calculation and implementation of L-edge XANES is currently being conducted by our research group. Furthermore, the analysis of XANES is recommended for the identification of oxidation state and coordination chemistry of the absorbing atom^30^. We note that the quantitative accuracy of XANES calculations is not comparable to EXAFS in identification of the distances, coordination number, and species of the neighbors of the absorbing atom. The accurate and precise interpretation of EXAFS is routinely conducted coupled with well-established software packages^31^ using the FEFF calculated EXAFS. The FEFF calculated K-edge EXAFS of all the materials available in the Materials Project database is underway, and a significant portion will be released in parallel with this publication.

## Additional information

**How to cite this article:** Mathew, K. *et al.* High-throughput computational X-ray absorption spectroscopy. *Sci. Data* 5:180151 doi: 10.1038/sdata.2018.151 (2018).

**Publisher’s note:** Springer Nature remains neutral with regard to jurisdictional claims in published maps and institutional affiliations.

## Supplementary Material



## Figures and Tables

**Table 1:** Keys and their description for each spectra data JSON file.

Key	Data Type	Description
*input_parameters*	string	the FEFF input settings used in the computation of the spectrum.
*xas_id*	string	unique id for each spectrum, e.g. 'mp-505011-28-XANES-K'.
*spectrum_type*	string	type of XAS e.g. 'XANES'.
*edge*	string	absorption edge e.g. 'K'.
*mp_id*	string	mp id of the structure.
*absorbing_atom*	string	site index of the absorbing site in the structure.
*structure*	string	the structure in dictionary format (can be loaded as a Structure object in pymatgen).
*spectrum*	float	array of shape (100, 6) where each column means the following(in that order):*Energy(eV), Energy with respect to the Fermi level(eV), Wave number, μ(total absorption coefficient), μ*_0_*(the background absorption coefficient), x(normalized finestructure)*
